# Electrochemical Study and Characterization of an Amperometric Biosensor Based on the Immobilization of Laccase in a Nanostructure of TiO_2_ Synthesized by the Sol-Gel Method

**DOI:** 10.3390/ma9070543

**Published:** 2016-07-07

**Authors:** Mariana Romero-Arcos, Ma. Guadalupe Garnica-Romo, Héctor Eduardo Martínez-Flores

**Affiliations:** 1 Programa Institucional de Doctorado en Ciencias Biológicas, Universidad Michoacana de San Nicolás de Hidalgo, Santiago Tapia 403, col. Centro, Morelia, Mich. cp 58000, Mexico; mariana_ra81@hotmail.com; 2 Facultad de Ingeniería Civil, Universidad Michoacana de San Nicolás de Hidalgo, Santiago Tapia 403, col. Centro, Morelia, Mich cp 58000, Mexico; 3 Facultad de Químico Farmacobiología, Universidad Michoacana de San Nicolás de Hidalgo, Santiago Tapia 403, col. Centro, Morelia, Mich cp 58000, Mexico; hedu65@hotmail.com

**Keywords:** sol-gel, amperometric biosensors, sensitivity, stability, detection limit

## Abstract

Laccase amperometric biosensors were developed to detect the catechol compound. The laccase enzyme (LAC) immobilization was performed on nanostructures of (a) titania (TiO_2_); (b) titania/Nafion (TiO_2_/NAF) (both immobilized by the sol-gel method) and a third nanostructure, which consisted of a single biosensor composite of Nafion and laccase enzyme denoted as NAF/LAC. The Nafion was deposited on a graphite electrode and used to avoid “cracking” on the matrix. The TiO_2_ particle size was an average of 66 nm. FTIR spectroscopy vibration modes of different composites were determined. The electrochemical behavior of the biosensor was studied using electrochemical spectroscopy (EIS) and cyclic voltammetry (CV). The biosensor based on TiO_2_/NAF/LAC presented the best electro-chemical properties with regard to sensitivity, stability and detection limit after a period of 22 days.

## 1. Introduction

A great number of polyphenolic compounds are byproducts resulting from the large-scale manufacture of several products such as drugs, dyes, pulp, plastic, pesticides and antioxidants [1,2,3]. Some of these phenolic compounds have been related to both health problems and environmental pollution, due to their inherent toxicity [3,4]. However, they have a significant antioxidant role with regard to the prevention of cardiovascular diseases and it is claimed that some may even prevent cancer, the latter being free radical scavengers, neutralizing oxygen reactive species and chelating metal ions [5,6]. Most analytic methods used in qualitative and quantitative determination of polyphenols, including ultraviolet spectrophotometry, gas chromatography (GC-MS), liquid chromatography (HPLC), or capillary electrophoresis (CE) [6,7,8,9], are time-consuming, require tedious sample preparation and expensive equipment, and may not be suitable for in situ monitoring [1,4,5,8,9,10]. Consequently, there is demand for a new analytical technique in order to determine low polyphenol concentrations.

Due to their good reproducibility, selectivity, high sensitivity, and miniaturization effect, electrochemical techniques, notably amperometric biosensors, stand out as the best candidates for the detection of polyphenols [1,6,8,9,10,11,12,13].

In recent years, biosensors, modified with oxidoreductase enzymes (tyrosinase, laccase, peroxidase or cellobiose dehydrogenase), have been developed for the detection of phenolic compounds, since phenols can act as electron donors for said enzymes [11,14,15,16].

One possible enzyme for the development of biosensors for phenols and polyphenols is laccase (EC 1.10.3.2). Laccase (EC 1.10.3.2), a multicopper redox enzyme widely occurring in fungi and less frequently in higher plants and bacteria, has been identified as a potential catalyst for the determination of phenolic compounds. The construction of the laccase biosensor is simple, as laccase does not require H_2_O_2_ as a co-substrate or any other external co-factor for its catalysis. Moreover, thermal tolerance is also a positive feature of laccase for biosensor applications. Laccase catalyzes the oxidation of various phenolic compounds with a concomitant reduction of molecular oxygen to water, without the intermediate formation of toxic H_2_O_2_. In general, laccase contains four copper atoms classified into type 1 (T1), type 2 (T2), and type 3 (T3), according to their spectroscopic and magnetic properties. T1, the substrate-binding site of the enzyme, is involved in the oxidation of the substrate and the transfer of electrons to the T2/T3 cluster, while T3 is responsible for oxygen uptake and, as a whole, the T2/T3 site is responsible for the reduction of O_2_ to water [9,11,14,15,16,17,18]. Enzyme immobilization is the key step in the fabrication of a sensitive and stable biosensor. Many methods (physical adsorption or covalent immobilization) have been developed to establish laccase-based biosensors [17,18,19].

In order to improve the characteristics of biosensors, a variety of functional materials have been used, such as Fe_3_O_4_ nanoparticles, chitosan, silica sol-gel, polypyrrole, Au-polypyrrole nanocomposites, silica, alumina and titania sol-gel via physical adsorption [1,10,19], either by cross-linking with, e.g., glutaraldehyde, or protecting with a thin gel/polymer layer of, e.g., Nafion to prevent the loss of enzymes [19].

The search for a novel immobilization system, together with an advanced material used to immobilize laccase in amperometric biosensor construction, continues to fascinate those working in the field. In recent years, sol-gel materials have emerged as one type of potentially attractive matrix, well suited for the said immobilization of enzymes in biosensor construction [8,10,11,20,21,22]. Sol-gel technology provides a unique means for preparing a three-dimensional network suitable for the encapsulation of a variety of biomolecules, because they can be prepared under ambient conditions, and they exhibit tunable porosity, high thermal stability and chemical inertness, plus they demonstrate negligible swelling in an aqueous solution. For these reasons, electrochemical biosensors, consisting of nanomaterials, have been extensively investigated and used in important industrial sectors, such as pharmaceuticals, health, food, agriculture and the environment. They provide real-time data, which allows the control and traceability of each process involved. TiO_2_ film has also been used for the immobilization of proteins for bio-analytical applications, given its stability and biocompatibility, and also because the biosensor based on laccase is immobilized in the titania gel matrix and prepared by the sol-gel method, both of which are attractive matrixes, prepared at room temperature, and can retain the catalytic activities of enzymes [8,10,11,23,24,25,26,27,28]. Some biomolecules trapped in the titania sol-gel matrix for the development of electrochemical biosensors have been reported. Zhang et al. developed a hybrid inorganic-organic material from titania, produced by sol-gel for the construction of a biosensor, immobilizing the tyrosinase enzyme, for the amperometric detection of phenolic compounds [29]. Lee et al. described a biosensor using tyrosinase placed in a composite film of titania-Nafion–doped nanotubes on the surface of multi-walled carbon [23]. The large pore size of the titania-Nafion film provided excellent stability and fast response time, due to the good biocompatibility of the titania-Nafion composite. An enzyme glucose-oxidase immobilized on TiO_2_ nanocrystals was used to develop an electrochemical biosensor for glucose oxidation, as described by Sousa et al. [30]. On the other hand, Zhong et al. developed an electrochemical biosensor for the determination of H_2_O_2_ based on the electrostatic immobilization of the enzyme peroxidase with gold nanowires and TiO_2_ nanoparticles on a gold electrode [31]. For the detection of phenolic compounds in environmental water samples, Kochana et al. proposed an amperometric biosensor based on tyrosinase immobilized in titania sol-gel [32]. The enzyme glucose oxidase was immobilized on a compound made from mesoporous hydroxyapatite and mesoporous titanium dioxide which were then ultrasonically mixed with multi-walled carbon nanotubes to form a rough nanocomposite film for detecting glucose (Li et al., 2012) [33].

The objective of this study was to develop an amperometric biosensor based on the immobilized laccase enzyme in order to detect catechol in solution. Immobilization of laccase was carried out in the TiO_2_ matrix, which was synthesized by the sol-gel method, so as to preserve the activity of the enzyme. The particle size and vibration modes of different compounds were measured by infrared spectroscopy. This method was used to develop two different biosensors (TiO_2_/LAC, TiO_2_/NAF/LAC), which were then compared with NAF/LAC system for detecting catechol (prepared at room temperature in order to preserve enzyme activity). The lifetime of the biosensors was studied over a period of 22 days.

## 2. Materials and Methods

### 2.1. Reagents

Laccase (EC 1.10.3.2, 10 U·mg^−1^ from the mushroom *Trametes versicolor*, St. Louis, MO, USA), precursor TiO_2_ titanium isopropoxide and Nafion (perfluorosulfonated ion-exchange resin, 5% (*w*/*v*) solution of 90% aliphatic alcohol and 10% water mixture), were purchased from Sigma-Aldrich (St. Louis, MO, USA). All chemicals were analytical grade. Solutions were prepared using de-ionized water. Graphite electrodes (ϕ = 3 mm) were from material grade: ST-21, Bay Carbon, INC. (Bay City, MI, USA) The supporting electrolyte was phosphate buffer 0.1 mol·L^−1^ (PBS) prepared with Na_2_HPO_4_ and KH_2_PO_4_ at natural pH of the solution. Catechol were purchased from Sigma-Aldrich.

### 2.2. Apparatus

Analyses were performed on an Infrared Spectroscopy Fourier Transform Infrared (FTIR). Spectra of liquid samples were recorded in the range of 500−4000 cm^−1^ on FTIR/ATR (Vertex 70, Bruker, Billerica, MA, USA).

Electrochemical measurements such as Cyclic Voltammetry (CV) and Electrochemical Impedance Spectroscopy (EIS) were performed with a Potentiostat/Galvanostat/ZRA, Gamry Instruments, Inc., (Philadelphia, PA, USA). All experiments were carried out with a conventional three-electrode system in a 40 mL electrochemical cell, working with electrode graphite, coated with different composites. The counter electrode was a platinum wire. A Saturated Calomel Electrode (SCE) was used as a reference. All experiments were performed at room temperature.

### 2.3. Preparation of Titania Sol

The titania was prepared by the sol-gel method, as recommended by Kochana et al. [10], and subsequently modified. Then 2 mL of titanium isopropoxide was added to 10 mL of 2-propanol acidified with 80 μL of concentrated CH_3_COOH and 160 μL of concentrated HNO_3_ and then stirred. The precursor was added extremely slowly to 24 mL of cold water being constantly stirred. The prepared sol was aged for 15 days, before being used for the preparation of biosensors.

### 2.4. Preparation of Biosensor

Prior to the fabrication of biosensors, the graphite electrode (GE) was carefully and repeatedly polished with metallographic abrasive and slurries of 0.3 and 0.05 µm alumina powder, until it acquired a mirror finish. After being thoroughly rinsed in double distilled water, the electrode was sonicated with 0.1 mol/L HCl, absolute ethanol, followed by de-ionized water for 5 min, respectively, and finally allowed to air-dry.

Then 20 µL titania sol (TiO_2_) prepared with 40 µL Nafion was stirred for 60 s in a Vortex, then, a solution of laccase (20 mg/mL) in phosphate buffer solution (pH = 6.80, 0.1 M) was prepared, mixed and sonicated with the composite of the titania sol with/without NAF, subsequently referred to as TiO_2_/NAF/LAC and TiO_2_/LAC, respectively. Likewise, one composite laccase/Nafion was carried out in 1:1 (*v*/*v*) referred to as NAF/LAC, 23 μL of the prepared mixtures were deposited on the pretreated surface of the electrode. The enzymatic biosensors manufactured were finally allowed to air-dry overnight and washed with de-ionized water before use. The enzymatic electrodes were stored at 4 °C until required.

## 3. Results and Discussion

### 3.1. Particle Size

The particle size distribution of titanium dioxide, which exhibits a very uniform distribution, presents a narrow range, concentrated in approximately 12 to 96 nm, and the size average is 66 nm.

### 3.2. FTIR Spectroscopy

**Figure 1:**
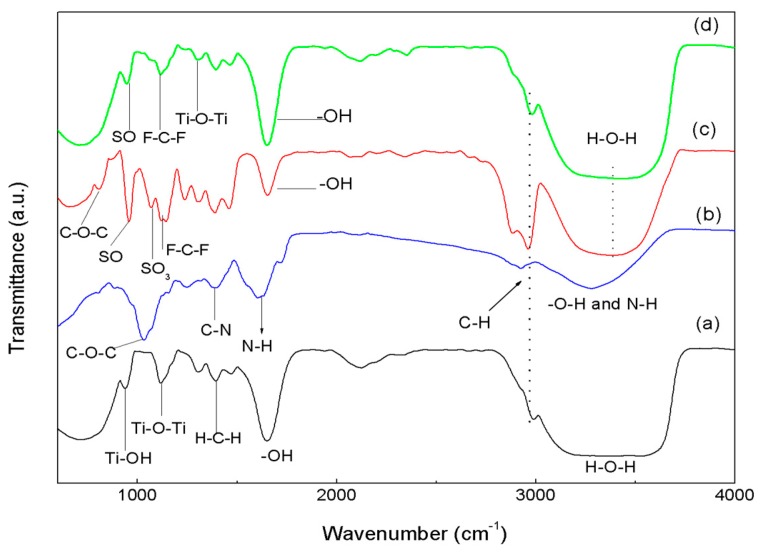
FTIR spectrum of (**a**) TiO_2_; (**b**) laccase; (**c**) Nafion; (**d**) TiO_2_/NAF/LAC.

The results of FTIR spectroscopy, with compositions of (a) Sol-TiO_2_; (b) pure laccase; (c) Nafion; and (d) TiO_2_/NAF/LAC composite, are shown in Figure 1. We can see that the bands at 3400 and 1620 cm^−1^ of the spectrum Sol-TiO_2_ (see (a)) result from the stretching and bending of the O–H group. The band at 692 cm^−1^ shows the stretching vibration of Ti–O [24]. However, those of 933 cm^−1^ and 1126 cm^−1^ are characteristic of the formation of the O–Ti–O network [25,26]. The band centered at 1301 cm^−1^ corresponds to the C–O stretch bond present in residual organics [34]. This band is a result of the adsorbed organic terminal titanium alkoxide (–OC_3_H_7_) incorporated into the titanium structure. The band at 2973 cm^−1^ is due to the C–H stretch from the presence of alkyl groups [26]. The doublet in 1392 cm^−1^ and 1538 cm^−1^ designates the symmetric and asymmetric stretching vibration of the carboxylic group coordinated by Ti, as a bidentate ligand.

The FTIR spectrum typical laccase (see (b)) shows five main bands: (1) a strong band centered at 3300 cm^−1^ due to O–H and NH vibrations; (2) weak bands at around 2800 cm^−1^ attributed to CH bonds; (3) a band corresponding to the NH peptide linkage at 1630 cm^−1^; (4) a band peaking at 1350 cm^−1^ attributed to the CN stretching vibration of amines; and (5) a strong and sharp band peaking at 1100 cm^−1^ due to COC [35,36,37,38].

However, the FTIR spectrum (see (c)) of Nafion presents links of a polytetrafluoroethylene backbone as hydrophobic by nature which produces two strong absorption bands at 1214 and 1155 cm^−1^; these bands are assigned to the asymmetric and symmetric stretching of bond C–F. Band doublets 829 and 983 cm^−1^, plus the band at 1053 cm^−1^, are characteristic of the Nafion side-chain, assigned to the symmetric vibration of links C–O–C and S–O and the stretching vibration of the groups SO_3_–, respectively. Furthermore, two bands characteristic of the presence of water molecules located at 3396 and 1640 cm^−1^ are present [39].

Finally, the FTIR analysis of TiO_2_/NAF/LAC (see (d)) presents the main link bands Ti–O–Ti, Ti–O–H, and C–F, S–O, mentioned above, thereby proving that the composite had been successfully prepared and the addition of titanium particles did not affect the chemical groups of the enzyme, and effectively demonstrating the biocompatibility of TiO_2_.

### 3.3. Electrochemical Impedance Spectra (EIS) Characterization of the Biosensor

Electrochemical Impedance Spectra (EIS) is a powerful tool for studying the interface properties of surface-modified electrodes. The impedance measurements of GE, GE-NAF/LAC, and GE-TiO_2_/LAC, GE-TiO_2_/LAC 1:1 and GE-TiO_2_/LAC 1:2 electrodes were carried out at the open circuit potential in phosphate buffer (pH 6.68) containing 1 mM K_3_[Fe(CN)_6_]/K4[Fe(CN)_6_] in the presence of 0.1 M KCl at a frequency range of 100 mHz to 100 KHz.

The curve of EIS presented as “the Nyquist plot” consists of two parts: first is the semicircular section, located at higher frequencies corresponding to the electron-transfer limited process. (The electron-transfer resistance (R_ct_) can be obtained by measuring its diameter.) The second is the linear section, which delivers information related to the diffusion process, held in the solution and located at lower frequencies.

**Figure 2:**
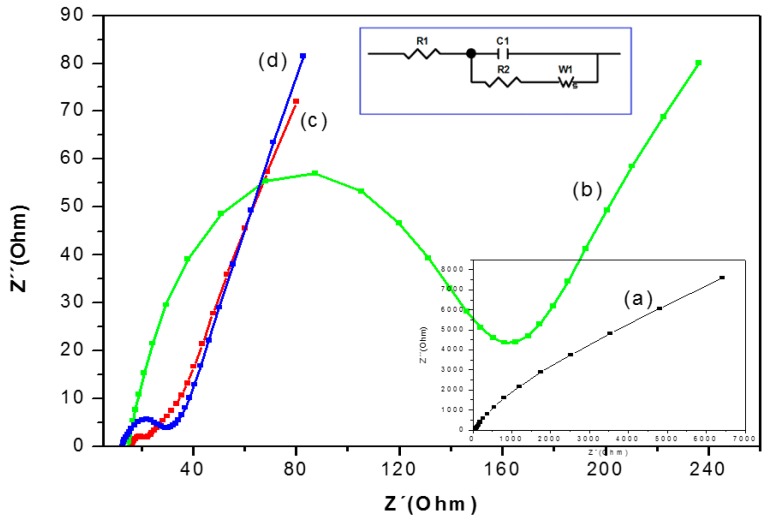
The Nyquist plots for (**a**) bare graphite (GE); (**b**) electrode NAF/LAC; (**c**) electrode TiO_2_/LAC; and (**d**) TiO_2_/NAF/LAC in the presence of 0.1 M KCl solution containing 1 mM K_3_Fe(CN)_6_/K_4_Fe(CN)_6_ (1:1) by applying an AC voltage with 50 mV amplitude at a frequency range of 100 mHz to 100 KHz.

This second section is used to analyze the detailed electrochemical response of the modified electrodes by employing individual or mixed Nyquist components [40,41,42]. The graphite electrode (GE) shows a linear behavior in the Nyquist spectra (see insert Figure 2), which is characteristic of a limiting step in the electrochemical process.

The semi-circle at high frequencies appears when the deposition of the composite converts to NAF/LAC (Figure 2b); this increase is attributable to the non-conductive properties of the laccase enzyme which obstructs electron transfer (143.40 Ohm) in the electrochemical cell [40,41,42,43]. Diameter reduction of the semi-circle was obtained with the composite TiO_2_/LAC, resulting in decreased resistance to the charge transfer (16.03 Ohm) at the interface of the electrode (Figure 2c). This increased charge transfer on the electrode surface is caused by the monolayer of TiO_2_, due to its semi-conductive properties.

Finally, it can be appreciated that the adsorption of laccase in the mixture of TiO_2_/NAF/LAC (Figure 2d) is directly related to the decrease of the semi-circle and the increased electron transfer (9.44 Ohm) due to rapid diffusion/migration in the film made with TiO_2_/NAF as compared to the NAF/LAC film. Moreover, the decrease in the semi-circle at high frequency adsorption of the laccase enzyme in the composite TiO_2_/NAF is due to the properties of moderate hydrophobicity presented by Nafion, which is necessary to maintain optimum activity of the enzyme. Because the Nafion has a hydrophobic fluorocarbon skeleton and site of hydrophilic cation exchange, when Nafion is mixed with the sol-gel titania, it forms an organic-inorganic hybrid matrix immobilization of laccase; similar results have been reported by Lee et al. (2007) and Kochana (2012) [23,44].

### 3.4. Cyclic Voltammetry (CV)

In this study, cyclic voltammetry (CV) was applied to illustrate electron transfer with reference to this multicopper enzyme, the graphite surface and the various modified electrodes, without changing the accepted technique.

This experiment was carried out in a solution of [Fe(CN)_6_]^3−^/[Fe(CN)_6_]^4−^ 1 mM dissolved in 0.1 M KCl at a scan rate of 100 V/s at a range of −0.5 to 0.70 V for the unmodified graphite electrode (GE), as well as with biosensors doped with TiO_2_.

**Figure 3:**
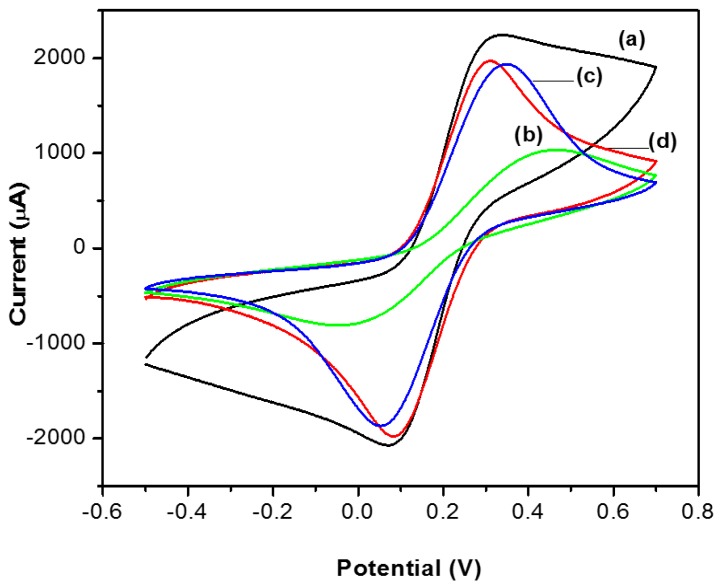
CVs of: (**a**) graphite (GE); (**b**) biosensor NAF/LAC; (**c**) biosensor TiO_2_/LAC; and (**d**) biosensor TiO_2_/NAF/LAC in 0.1 M KCl solution containing 1 mM K_3_Fe(CN)_6_/K_4_Fe(CN)_6_ (1:1), at a scan rate of 100 mV/s.

In Figure 3, well-defined peaks can be seen for the transducer of graphite (Figure 3a) and for the biosensors TiO_2_/NAF/LAC and TiO_2_/LAC (b and c), which are attributable to the condition of graphite and the semi-conducting properties of TiO_2_. By contrast, the biosensor NAF/LAC (d) has a very small current peak in the cyclic voltammogram, due to the Nafion being partially surrounded by hydrophobic regions of sulfonate perfluorinated ionomer, which hinders the ion transfer and non-conductive properties of the enzyme as reported in [31,44]. In addition, it is seen that the TiO_2_/NAF/LAC and TiO_2_/LAC biosensors exhibit less reversibility and better response in the peak current than the electrode NAF/LAC.

This behavior can be attributed to the laccase which remained adsorbed in the titania matrix, and therefore did not obstruct the passage of electrons from the electrochemical probe which is consistent with the results of the electrochemical impedance technique [45].

### 3.5. Cyclic Voltammetry Behavior of Catechol

**Figure 4:**
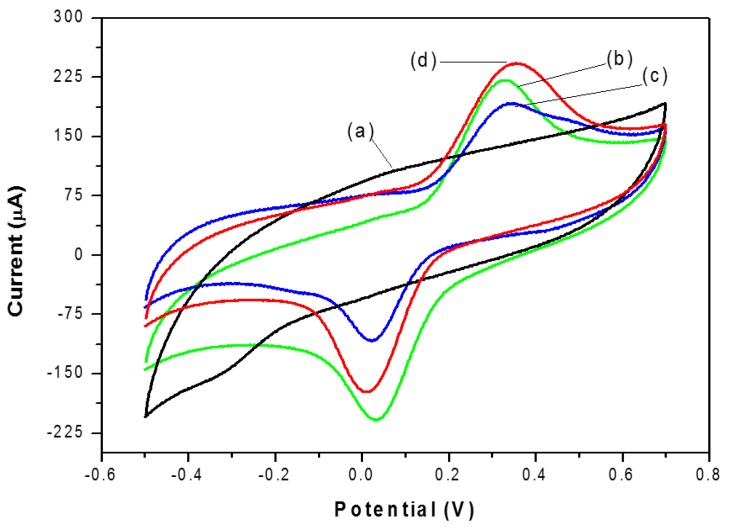
Shows cyclic voltammograms of: (**a**) GE; (**b**) NAF/LAC; (**c**) TiO_2_/LAC; and (**d**) TiO_2_/NAF/LAC in the presence of 40 mM catechol in 0.1 M SBP (pH 6.68) at a scan rate of 100 mV.

**Figure 5:**
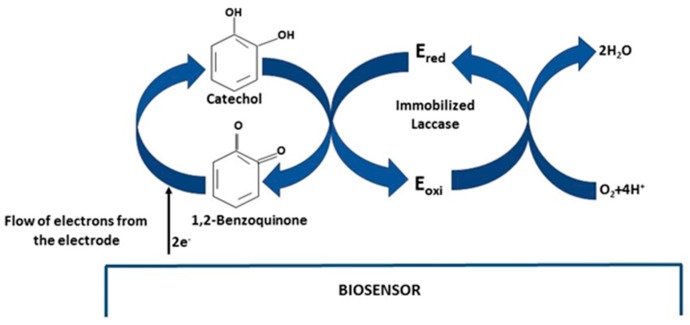
Electron transfer mechanism of catechol oxidation on a laccase biosensor. E_red_ = reduced enzyme and E_oxi_ = oxidized enzyme.

Figure 4 displays the typical cyclic voltammograms of catechol (40 μM) in pH 6.68 PBS on the graphite electrode (curve a); NAF/LAC (curve b); TiO_2_/LAC (curve c) and TiO_2_/NAF/LAC (curve d). Curve a exhibits the behavior of the graphite electrode. As expected, there are no redox peaks observed in the absence of laccase, even at a concentration of up to 40 μM of catechol. Nevertheless, a significant increase is seen in the current peak reduction, when the graphite electrode is modified with laccase (curves b, c and d). This increase in the reduction peak follows the direct lowering of o-quinone, producing the reaction catalyzed by the enzyme on the surface of the electrode [3,6,46]. The peak current reduction of catechol in TiO_2_/NAF/LAC is higher, proving that TiO_2_ particles play an important role in the transfer of electrons between o-quinone and the electrode surface, as a result of the semi-conductive properties. Such detection is based on the enzymatic oxidation reaction and the subsequent amplification cycle of the recycling of the substrate/analyte on the electrode. The mechanism reaction is presented in Figure 5, in which the catechol reacts with LAC, and is oxidized to 1,2-benzoquinone in presence of O_2_. Subsequently, 1,2-benzoquinone is electrochemically reduced at appropriate potentials on the biosensor surface. Therefore, the peak current obtained in the process of the electrochemical reduction of 1,2-benzoquinone to catechol is proportional to the concentration of catechol. [7,14,15,47,48,49].

### 3.6. Amperometric Response of Catechol

**Figure 6:**
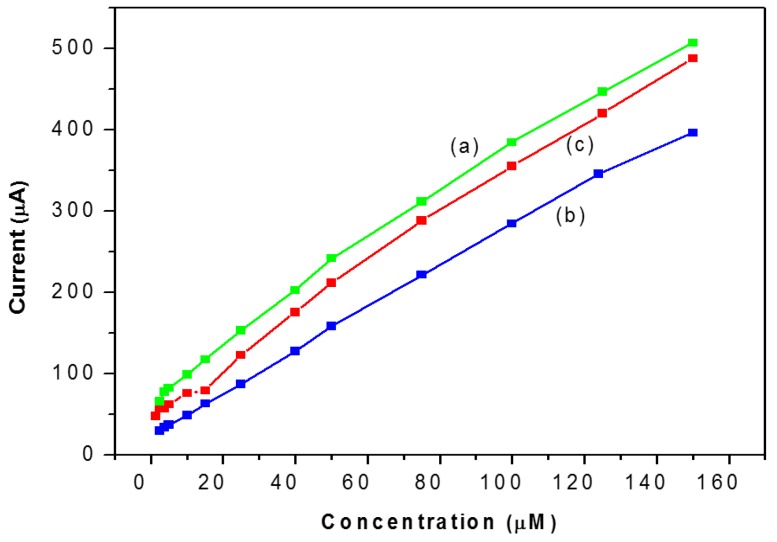
Calibration curve of the biosensor as a function of the concentrations of catechol: (**a**) NAF/LAC; (**b**) TiO_2_/LAC; and (**c**) TiO_2_/NAF/LAC.

The electrochemical responses of the NAF/LAC, TiO_2_/LAC and TiO_2_/NAF/LAC biosensors have been investigated as a function of the concentration of catechol (from 5–150 µM) using the technique of CV with a scan rate 100 mV/s, in PBS (50 mM, pH 6.68). In studies of bioelectrode response, it was observed that the magnitude of the current response increases according to the rising concentration of catechol (Figure 6).

Based on biosensors NAF/LAC and TiO_2_/LAC (curves a and b), these exhibit the same behavior in terms of detection limit 2.5 μM, linear range (1.25–150) and a correlation factor of 0.9944 and 0.9986, respectively. Furthermore, the biosensor TiO_2_/NAF/LAC presents the best electrochemical response, with a detection limit of 0.75 μM, a wide linear range of 0.75 to 150 μM, and a correlation factor of 0.9966. Similarly, the sensitivity of biosensors was calculated, resulting in 2.6, 2.71 and 2.94 μA/μM to NAF/LAC, TiO_2_/LAC and TiO_2_/NAF/LAC, respectively.

The sensitivity, linear range and detection limit of the enzyme biosensors were tested on the first, seventh, 15th and 22nd days. See the listed results in Table 1 which demonstrate that based on the composite biosensor, the sensor made with TiO_2_/Nafion/laccase presented a 13% higher sensitivity compared to the sensor prepared with Nafion/laccase. This result was due to the inherent biocompatibility of sol-gel–derived titania film and indicative of the biocompatibility of titania/Nafion hydrogel with the enzyme, rendering a substantial improvement in the long-term stability of the biosensor. Additionally, the network structure of the titania/Nafion composite matrix can prevent leaching of the enzyme, which favors the stability of this biosensor [50].

### 3.7. Biosensor Stability

The reproducibility of the biosensors based on NAF/LAC, TiO_2_/LAC and TiO_2_/NAF/LAC was evaluated through the detection of 40 µM in 0.1 M PBS (pH 6.68) when six successive determinations were made. A relative standard deviation (RSD) of 7%, 9% and 7% was obtained for the biosensors made with NAF/LAC, TiO_2_/LAC and TiO_2_/NAF/LAC, respectively, demonstrating that the modifications of the electrodes allow for good reproducibility during measurements, without applying a pretreatment to the biosensors. Moreover, long-term stability is considered one of the key factors in the performance of the biosensors, which is evaluated by measuring the response of 10 µM catechol for 22 days. When the electrodes were not used, they were stored at 4 °C in dry conditions, based on the evidence that the enzymatic biosensors retained their activity at 92%, 87% and 94% for the biosensors made with NAF/LAC, TiO_2_/LAC and TiO_2_/NAF/LAC, respectively, after a week. After 22 days, biosensors made with NAF/LAC, TiO_2_/LAC and TiO_2_/NAF/LAC retained activities of 58%, 53% and 69%, respectively, indicating good stability of the biosensors, especially the biosensor based on TiO_2_/NAF/LAC, which shows that the film of titania sol-gel is very efficient in retaining laccase activity.

## 4. Conclusions

The results presented in this work demonstrate that the three biosensors (TiO_2_/LAC, NAF/LAC and TiO_2_/NAF/LAC) developed in this research have a good level of sensitivity towards catechol in the order of µA/µM when compared to those reported by other authors in the field who obtained results of µA/mol. By far the best analytical properties were seen in the biosensor TiO_2_/NAF/LAC. This is due to the semi-conductive properties of TiO_2_ together with the proposed sol-gel method, which provided a non-aggressive immobilization process of the laccase enzyme, and provided a surrounding biocompatible microenvironment by which it could fulfill its biological activity.

## Figures and Tables

**Table 1:** The sensitivity, linear range and detection limit of enzyme biosensors were tested on the first, seventh, 15th and 22nd days.

Days	Analytical Characteristics	NAF/LAC	TiO_2_/LAC	TiO_2_/NAF/LAC
1	Sensitivity (µA·L/µmol)	2.6	2.71	2.94
	Linear Range/µM	1.25–150	1.25–150	0.75–150
	Detection limit/µM	1.25	1.25	0.75
	*R*^2^	0.9944	0.9986	0.9966
7	Sensitivity (µA·L/µmol)	2.78	2.62	2.85
	Linear Range/µM	2.5–125	2.5–150	1.25–150
	Detection limit/µM	2.5	2.5	1.25
	*R*^2^	0.951	0.9962	0.9988
15	Sensitivity (µA·L/µmol)	2.6	2.4	2.8
	Linear Range/µM	5–150	15–150	1.25–100
	Detection limit/µM	5	3.75	1.25
	*R*^2^	0.9993	0.9958	0.9966
22	Sensitivity (µA·L/µmol)	2.12	1.92	2.5
	Linear Range/µM	5–150	3.75–150	3.75–150
	Detection limit/µM	5	3.75	3.75
	*R*^2^	0.9973	0.9988	0.9980

## Abbreviations

The following abbreviations are used in this manuscript:

FTIR	Fourier Transform Infrared
CV	Cyclic Voltammetry
EIS	Electrochemical Impedance Spectroscopy
SCE	Satured Calomel Electrode
GE	Graphite Electrode
NAF	Nafion
LAC	Laccase
TiO_2_	Titania sol
